# Psychological Profile and Distinct Salivary Cortisol Awake Response (CAR) in Two Different Study Populations with Obstructive Sleep Apnea (OSA) and Central Serous Chorioretinopathy (CSC)

**DOI:** 10.3390/jcm9082490

**Published:** 2020-08-03

**Authors:** Fabio Scarinci, Francesca Romana Patacchioli, Cristina Mihaela Ghiciuc, Vittorio Pasquali, Raluca Mihaela Bercea, Sebastian Cozma, Mariacristina Parravano

**Affiliations:** 1 IRCCS—Fondazione Bietti, 00100 Rome, Italy; fabioscarinci@gmail.com (F.S.); mcparravano@gmail.com (M.P.); 2 Department of Pharmacology, School of Medicine, University of Medicine and Pharmacy “Grigore T. Popa”, 700115 Iaşi, Romania; c_ghiciuc@yahoo.com; 3 Department of Psychology, Sapienza University of Rome, 00100 Rome, Italy; vittorio.pasquali@uniromauno.it; 4 Department of Pneumology, County Emergency Hospital of Ploieşti, 100248 Ploieşti, Romania; ralbercea@yahoo.com; 5 Department of Otorhinolaryngology, School of Medicine, University of Medicine and Pharmacy “Grigore T. Popa”, 700115 Iasi, Romania; scozma2005@yahoo.com

**Keywords:** salivary cortisol, AUC_CAR_, SLOPE_CAR_, obstructive sleep apnea (OSA), central serous chorioretinopathy (CSC), Daily Hassles and Stress (DHS) questionnaire, Hamilton Rating Scale for Depression (HAD), Hamilton Anxiety Rating Scale (HAS)

## Abstract

Obstructive sleep apnea (OSA) and central serous chorioretinopathy (CSC) are in terms of nosography different pathologies, however they share a stress-related physio-pathogenetic component, not yet explored in depth. Therefore, the aim of the present study was to ascertain whether OSA and CSC share a common profile, specifically in cortisol production focusing on the cortisol awake response (CAR), the area under curve (AUC_CAR_) and the SLOPE_CAR_ compared with healthy matched controls. Furthermore, standardized self-administered questionnaires were used to identify mental health status related to depression, anxiety and subjective stress perception levels in the study populations. The results showed hypothalamus-pituitary-adrenal (HPA) axis activity anomalies, represented by a flattening CAR in the OSA group and a statistically significant increase in cortisol production in CSC patients at awakening. This disarrangement of the HPA axis activity associated with elevated distress and mental health scores, and its presence in both patients with OSA and patients with CSC, might represent the shared path explaining the stress-related component in these diseases. Further research is needed to investigate the psycho-neuro-endocrinological aspects of OSA and CSC to determine whether psychoeducation on effective stress coping strategies might be of value in improving the quality of life of OSA and CSC patients.

## 1. Introduction

The pathogenic role of obstructive sleep apnea (OSA), characterized by repetitive upper airway occlusion episodes leading to apnea and excessive daytime sleepiness [1,2,3], is increasingly accepted in cardiovascular and cerebrovascular diseases [4].

In selected patients, the local and systemic consequences of OSA might contribute to the occurrence and/or the aggravation of the ocular pathologies. OSA has been associated with several ocular diseases, including glaucoma, nonarteritic anterior ischemic optic neuropathy and retinal vein occlusion [5,6]. Furthermore, OSA has been considered an important risk factor for developing central serous chorioretinopathy (CSC) [7], which is characterized by serous detachment of the neurosensory retina, occurring most frequently in mid-life and more often in men than in women [8,9,10]. Particularly, sleep fragmentation and deprivation, hypercapnia, vascular endothelial dysfunction, platelet aggregability and last, but not least, dysregulation of sympathetic activation, with increased levels of circulating epinephrine and norepinephrine may play an important role targeting the retinal microcirculation [11,12,13,14].

Since autonomic activation can stimulate hypothalamic-pituitary-adrenal (HPA) axis activity [15,16] and nocturnal awakenings have been related to pulsatile cortisol release [17], OSA would be expected to activate the HPA axis [18]. Apneic events, both directly and indirectly, can lead to increased cortisol levels by disrupting the hormone regulatory response and activating the HPA axis [5,19].

Among the various risk factors recognized for CSC, there is exposure to increased levels of endogenous or exogenous glucocorticoids [20,21,22].

The altered cortisol production potentially represents one pathophysiological link between OSA and CSC. However, unassessed pathophysiological pathways linking OSA and CSC cannot be totally excluded. Indeed, recently, Schellewis et al. identified genes involved in the control of tumor necrosis factor alpha levels among individuals with CSC [23]. In parallel, Imani et al. showed altered serum and plasma tumor necrosis factor levels among individuals with OSA [24].

Behavioral characteristics and psychosocial stress have traditionally been associated with both OSA and CSC, considered separately and as mutual comorbidities [20,25,26].

Psychosocial distress has been largely studied in patients diagnosed with OSA: symptoms associated with OSA include difficulty concentrating, cognitive impairment, depression and a general decrease in daytime quality of life [27,28]. An interesting report by Ohayon [29] using the ‘‘Sleep-EVAL’’ expert system in telephone interviews shows in a population survey among 857 people identified as having OSA, the prevalence of major depressive disorder of about 17% in comparison with 4.3% in healthy subjects. In a retrospective study of US Veterans Health Administration 118,105 patients among more than 4 million subject records had physician-diagnosed OSA and among these, 21.8% were diagnosed with depressive disorder (three times the prevalence measured in patients without OSA) [30].

In 167 patients diagnosed with OSA attending a Dutch sleep clinic, Vandeputte et al. found that 41% of those patients had a Beck Depression Inventory score of 10 or more, indicating the probable presence of depression [31]. Hayashida and coworkers studied 230 OSA subjects showing that subjective daytime sleepiness in patients with OSA may be influenced by certain personality characteristics affecting the hypochondriasis score [32]. Pierobon et al. found that OSA patients had a higher frequency of extroversion and depressive behaviors as well as impairment of memory and cognitive function in these patients [33].

However, it should be considered that any association between OSA and depressive symptoms might be influenced by uncontrolled confounding factors including obesity, age, gender and hypertension.

Patients with CSC revealed a higher tendency to present schizophrenia, hysteria, depression, psychopathic deviance, hypochondriasis, higher levels of frustration and anticipatory anxiety than control groups [34,35]. In addition, higher anxiety scores were also observed in Iranian patients with CSC [36].

The clinical course of CSC has also been attributed to stress, psychosocial factors and lifestyle [37]. In comparison with healthy controls, it has been shown that CSC patients have a significantly higher degree of emotional distress [38,39,40] and more marked psychological symptoms associated with poorer quality of life [41].

Based on the literature selection above reported, we can expect that OSA and CSC can share their stress-related component, presenting common neuroendocrine and psychological asset derangements. However, the identification of common stress-related etiopathogenetic processes has not yet been clarified conclusively and the available studies do not provide clear evidence of a common feature between OSA and CSC in the production of cortisol, the major end product of the HPA axis.

Therefore, the aim of the present study was to ascertain whether OSA and CSC shared a common profile specifically in the cortisol awake response (CAR), which reflects changes in the cortisol concentrations that occur during the first 30 min after awakening from night sleep and is currently used to assess HPA axis activity under different physio-pathological conditions.

Therefore, this study was designed to determine

(a) the magnitude of the CAR by measuring the integrated volume of cortisol released over 30 min of the waking period (AUC_CAR_); and
(b) the slope of the wake-up response (SLOPE_CAR_), which means the incline of the straight line that passes through the two time points of the salivary cortisol measurements, made upon awakening and 30 min later

in two populations of adult male patients homogeneous in demographic and somatic characteristics and affected by OSA or CSC, in comparison with healthy controls.

Furthermore, the psychological profile of the study populations was evaluated by standardized questionnaires to assess patients’ mental health status in comparison with controls, using the Hamilton Rating Scale for depression (HSD) [42], the Hamilton Anxiety Rating Scale [43], and the Daily Hassles Scale (DHS) [44], measuring individual subjective stress perception induced by daily hassles.

## 2. Material and Methods

### 2.1. Study Populations

This retrospective study was conducted in accordance with the Declaration of Helsinki, and the protocol was formally approved by the local Ethics Committee (The Grigore T Popa University Institutional Review Board: Protocol No. 6832, 14 April 2020; the Clinic of Pulmonary Diseases Institutional Ethics Committe: Protocol No. 14, 29 April 2011—Iasi—Romania; the Central Ethical Committee for Lazio, Italy: Protocol No. 4327, 18 April 2018).

According to the a priori sample size calculation made by ClinCalc.com [45], 28 subjects for each group of the study populations (14 patients and 14 controls) were required to detect in the control groups a mean absolute difference of approximately 70% for the expected changes in salivary cortisol concentrations between the values measured at awakening and 30 min thereafter to have enough statistical power to detect a possible difference between groups (alpha = 0.05; beta = 0.2; post hoc power = 81.5%).

#### 2.1.1. OSA Study Population

The OSA study group included 14 Caucasian male subjects consecutively attending the Centre for Sleep Disturbances of the Clinic of Pulmonary Diseases between April 2011 and December 2015. They were 40 to 60 years old, with a body mass index (BMI) <30 kg/m^2^ [46], newly diagnosed with severe OSA based on nocturnal polysomnography (PSG) evaluation (apnea-hypoapnea index, AHI ≥30 h^−1^) and excessive daytime sleepiness [47].

The exclusion criteria were as follows: acute or chronic associated diseases (in particular, any ocular diseases); smoking; use of any chronic medication; treatment with corticosteroids; immunosuppressive diseases; or missing signed informed consent.

Fourteen matched healthy controls were enrolled among subjects accompanying patients to the Centre for Sleep Disturbances who had the same somatic characteristics of the OSA patients; for which the same exclusion criteria for the OSA patients were followed and they did not suffer from any sleep-disordered breathing.

#### 2.1.2. CSC Study Population

Fourteen consecutive Caucasian male subjects who were aged 40 to 60 years, had a body mass index (BMI) < 30 kg/m^2^, and attended the outpatient clinic of Retina Medical Service at the Bietti Foundation were enrolled from May 2018 to March 2019. They were newly diagnosed with acute idiopathic CSC, presenting with a leakage at the level of the retinal pigment epithelium (RPE) and with subretinal fluid, confirmed respectively by fluorescein angiography and spectral domain-optical coherence tomography B-scan (SD-OCT) [48,49,50].

Occult choroidal neovascularization was excluded by indocyanine green angiography performed to explore choroidal vascular hyperpermeability [51]. Patients with chronic CSC or recurrent CSC, macular degeneration or diabetic retinopathy, uveitis history, optic disc edema, choroidal infiltrates, cotton wool spots or retinal hemorrhages were also excluded. Further exclusion criteria included the following: smoking; use of any chronic medication; treatment with corticosteroids in the year preceding the development of CSC; acute or chronic associated diseases (in particular, sleep-disordered breathing); immunosuppressive diseases; or missing signed informed consent.

Fourteen healthy matched controls were recruited among Bietti Foundation employers who did not present any ocular abnormalities as shown by SD-OCT, for which the same exclusion criteria for the CSC patients were followed.

### 2.2. Experimental Protocol

The same experimental protocol was strictly followed for the two study populations. In the present retrospective study, we enrolled males only because the synchronization of the menstrual cycle, necessary to avoid the confounding cortisol release modulation operated by female hormones in women, was not possible.

Written informed consent was obtained from all participants during a preliminary informative meeting. During a subsequent experimental session (always between 09:00 h and 12:00 h), upon arrival, the characteristics (height, weight and blood pressure measured by digital monitor) of the study population were collected.

Then, subjects were invited to complete self-administered questionnaires. The psychometric evaluations were performed with: (1) the Hamilton Rating Scale for Depression (HDS) [42], a 17-items scale some defined in terms of a series of categories of increasing intensity, while others are defined by a number of equal-valued terms; (2) the Hamilton Anxiety Rating Scale (HAS) [43], a 14-items scale measuring the severity of anxiety symptoms in which each item is scored on a 4-point scale of 0 (not present) to 4 (severe); (3) the Daily Hassles and Stress (DHS) questionnaire [44], a 51-items scale measuring individual subjective stress perception levels on a 4-point scale ranging from 1 (never) to 4 (very often).

At the end of the session, all study participants were instructed on how to collect saliva at home and were asked to avoid teeth brushing, food, coffee and any physical exercise before each saliva collection [52,53]. Home diurnal saliva collection was scheduled at naturally awakening (always between 6:30–7:30) and 30 min later, on a week day.

To enhance as much compliance as possible with respect to the exact time of saliva sampling, all participants were asked to annotate the time of collection on a preprinted sheet supplied together with the collection tubes and/or to send at each time point scheduled on the collection day a short-message service (SMS) to a staff member. The day after saliva collection at home, subjects returned the samples to the outpatient clinic.

### 2.3. Saliva Collection and Biomarker Assay

Saliva was collected using the Salivette sampling device (Sarstedt, Milan, Italy). The saliva was recovered by centrifugation at 3000 rpm for 15 min and then frozen at –20°C until the analysis was performed using commercially available assay kits and commercial immune-enzymatic kits (DiaMetra, Milan, Italy) for the direct salivary assay of salivary cortisol (the inter-assay coefficient of variation was <10%, and the intra-assay coefficient of variation was <7%, with a minimum detectable concentration of 0.5 ng/mL).

### 2.4. Statistical Analyses

All data are reported as the mean ± SE and (SD), unless otherwise specified. The statistical analyses and graphics were performed using the SigmaPlot-11 software package (SxST.it, Milan, Italy). The Kolmogorov–Smirnov statistic was used to test for normal distributions prior to statistical analyses.

The area under the curve (with respect to ground) of cortisol production in the first 30 min after awakening (AUC_CAR_) [54,55] and the slope of the wake-up response (SLOPE_CAR_) were calculated for each subject.

One-way Analysis of Variance (ANOVA) for continuous variables approaching a normal distribution, or a Kruskal–Wallis one-way ANOVA on Ranks for continuous non-normally distributed variables, was applied to analyze somatic and psychological variables as well as AUC_CAR_ and SLOPE_CAR_. Post hoc (Dunn, Holm-Sidak) method for multiple comparison was applied to reveal differences among study groups.

A two-way repeated measures (rm)ANOVA test was performed to reveal the salivary cortisol production at awakening. Post hoc analyses, using Bonferroni’s multiple comparison test, were conducted to reveal subgroup differences. The statistical significance was set at *p* < 0.05 [56].

## 3. Results

### 3.1. Characteristics of the Study Populations

All subjects of the study populations were Caucasian adult males. One-way ANOVA followed by post-hoc test for multiple comparisons reveals that there were no significant differences in the somatic and baseline clinical data among the control group participants, the OSA and the CSC patients. Furthermore, these data were within the normal ranges in all the study populations.

In particular, controls (*n* = 28) were 50 ± 3 years old with SD = 11, while OSA patients (*n* = 14) were 55 ± 2 years old with SD = 7 and the CSC patients were 48 ± 2 years old with SD = 8 (One-way ANOVA: F(_2, 53)_ = 2.373; *p* = 0.103; ns). The body mass index (BMI) was 25.1 ± 0.7 kg/m^2^ with SD = 2.7 for controls and 26.2 ± 0.4 kg/m^2^ with SD = 1.1 for OSA patients and 25.9 ± 0.5 kg/m^2^ with SD = 2.1 for CSC patients (One-way ANOVA: F_(2, 53)=_ 1.277 *p* = 0.103; ns). The systolic blood pressure (SBP) was 125 ± 3 mmHg with SD = 9 for controls and 126 ± 2 mmHg with SD = 6 for OSA patients, and 128 ± 1 mmHg with SD = 1.1 for CSC study population (One-way ANOVA: F_(2, 53)=_ 0.824 *p* = 0.444; ns). In addition, the diastolic blood pressure (DBP) was 75 ± 1 mmHg with SD = 4 for controls and 76 ± 2 mmHg with SD = 7 for OSA patients and 77 ± 2 mmHg with SD = 4 for the CSC population under study (One-way ANOVA: F_(2, 53)=_ 1.034 *p* = 0.363; ns). Finally, the heart rate (HR) was 72 ± 2 beats/min with SD = 6 for controls and 77 ± 3 beats/min with SD = 11 for OSA patients and 72 ± 1 beats/min with SD = 4 for CSC patients (Kruskal–Wallis One-way ANOVA on ranks: H = 1.621 with 2 degrees of freedom; *p* = 0.445; ns).

### 3.2. Psychological Profile in the Study Populations

As reported in Table 1, OSA patients showed slight but significantly higher scores on the HDS and HAS than the respective controls did. The CSC subjects showed significantly higher HDS scores than the controls did and showed no signs of anxiety. Furthermore, the HDS and HAS values reported in the control groups indicated the absence of signs of depression and anxiety. The DHS scale scores indicated, for OSA and CSC subjects, a medium level of perceived stress (scores between 76 and 115), and the scores were significantly higher than those of respective control group participants, who showed a nonpathological level of perceived stress (scores between 56 and 75 on the DHS scale).

### 3.3. Salivary Cortisol Production at Awakening in the Study Populations

The salivary cortisol concentrations measured in the study populations at awakening and 30 min after awakening are reported in Table 2.

The control group presented a typical CAR course, while the salivary cortisol concentration rose significantly 30 min after awakening. OSA subjects presented a loss of the CAR, since the salivary cortisol concentration did not increase 30 min after awakening. In contrast, CSC patients presented a typical CAR course, as seen from the significant increase in salivary cortisol levels measured 30 min after waking.

Two composite variables derived from the above-repeated salivary measurements were also calculated. Thus, the AUC_CAR_ with respect to ground was computed to estimate (Table 3) the total amount of salivary cortisol produced upon awakening and 30 min later (AUC_CAR_) as well as the slopes of the cortisol awake response (SLOPE_CAR_) derived from the equation of the straight line passing through the two time points of saliva collection: upon awakening and 30 min later.

A slight, statistically insignificant, reduction of about 15% was found between the AUC_CAR_ of the OSA subjects and that of the controls. In contrast, CSC patients showed a significantly higher (+47%) AUC_CAR_ than that of the controls.

In the OSA patient group, the SLOPE_CAR_ was almost flat and significantly different from that of controls and that of CSC group. In the CSC study population, the SLOPE_CAR_ of patients was not different from that of controls, indicating the expected incline of the straight line passing through the two time points of saliva collection at awakening.

## 4. Discussion

The present study highlights a blunted CAR and flattened SLOPE_CAR_ in OSA patients compared with those in healthy controls. In contrast, CSC patients showed the presence of the expected CAR morning peak, associated with a higher salivary cortisol concentration than that of controls at awakening and 30 min later.

Furthermore, the magnitude of the morning production of salivary cortisol follows two distinct trends in the study populations: during the first half hour of waking up, no changes have been detected in the amount of the AUC_CAR_ produced in OSA patients, whereas the CSC patients presented an enhanced AUC_CAR_ in comparison with that of the controls.

The aforementioned anomalies affecting salivary cortisol production at awakening, are consistent with HPA axis dysregulation in both OSA and CSC subjects who may share a common pathogenetic process in association with elevated subjective stress perceptions induced by daily hassles and significant HDS scores in comparison with healthy controls, while a moderate level of HAS is detectable in OSA patients only.

We have shown here that CAR, physiologically characterized by a peak of salivary cortisol production 30 min after awakening [54,57], was not detectable in the OSA population. Altered CAR has been described in psychiatric disorders [54,57,58,59,60] and in subjects suffering from stress-related illness [61,62] and fatigue-related symptoms [63].

HPA axis dysregulation is present in several stress-related diseases [64,65,66,67]. Indeed, the allostatic load model for stress by McEwen [68] originally speculated that a flatter pattern of cortisol secretion produced in response to repeated physio-pathological challenges reflects an alteration of HPA activity [68,69,70]. Thus, repeated apnea episodes might cause stressor-like alterations that induce dysregulation of the HPA axis in OSA patients [71].

According to our findings, Raff and coworkers reported no change in the morning concentration of salivary cortisol between OSA and controls [71]. In contrast, an enhanced secretion of cortisol has also been reported in obese hypertensive patients with OSA [72], in which dysregulations in HPA axis activity are linked to a range of OSA-induced negative health outcomes associated with hypercortisolemia [73,74]. It has also been shown that hypertension itself could impair HPA axis activity [75], and a reduced CAR has also been reported in obese subjects, regardless of the presence of OSA [73].

We have shown here that CSC involves a generalized overproduction of salivary cortisol compared with that observed in controls, both upon awakening and in the following 30 min. Since hypercortisolism has been reported to be associated with central obesity [76] and hypertension [77], to avoid the confounding effects of these comorbidities, the subjects enrolled in the present study had no signs of obesity (BMI < 30 kg/m^2^) or hypertension [47]. Furthermore, OSA and CSC study populations have homogeneous demographic profiles and include males aged 40–60 years or suffering from severe OSA with no visual disturbances or, conversely, patients with acute CSC with no sleep-disordered breathing.

As a whole, through measuring the magnitude the salivary cortisol produced upon awakening, we observed a dysregulation of the functional chronobiology of the stress system in both OSA and CSC patients, and we believe that this should be included among the trigger factors of some of the OSA and CSC stress-related features [22,78].

The magnitude of the AUC_CAR_, representing the integrated amount of salivary cortisol released over the waking period, has been related to a number of psychosocial factors [79].

Regarding psychological dimensions, we have shown that OSA patients score higher than controls do on depression and anxiety, while CSC subjects, who also present higher depression scores than controls do, did not differ compared to the control group in the psychopathological dimensions of anxiety.

Several studies have reported elevated depressive symptoms among OSA patients [80,81,82,83]. More recently, in line with our findings, it has been reported that OSA is a possible risk factor for developing depression [84]. Chen et al. showed with a longitudinal study that the incidence of depression was double in individuals with OSA, compared to those without OSA [85]. We have reported that in OSA the depressive state is associated with slight anxiety and mild stress, according with Nanthakumar and colleagues [86]. However, it should be noted that all the absolute depression and anxiety scores that we measured were almost borderline, which is lower than the value at which patients are normally referred for psychiatric care.

CSC is known to be related to anxiety and stress [36,39], and stress-induced hypercortisolism may contribute to the development of CSC [22,38,87]. Subjective stress perception has already been considered to be an important component of maladaptive personality traits, as well as psychosomatic comorbidity. Literature data report, in agreement with the present study, that the presence of emotional distress in CSC patients [39] is associated with depression [34]. However, higher levels of anticipatory anxiety have been detected in CSC patients [35] which is different to our findings, probably due to some differences in the characteristics of the study populations.

There are certain limitations to the present study, including its retrospective design, its relatively small sample size and its lack of longitudinal data. Studies with a larger sample size and a wider personality assessment are needed to confirm our findings. Furthermore, since the sampling of saliva for biomarker measurements occurred on only a single day, the day-to-day variability could not be taken into account [88].

In addition, in the present study, the HDS was used as a self-rating scale, while the HDS should be used lege artis as an expert’s rating scale.

Unfortunately, in the present study the association between psychological outcomes and cortisol was not examined: the sample size was too small to apply Spearman’s correlation analysis which requires a higher number of subjects in each group.

Finally, it is not well established whether symptoms of psychological distress are related to OSA and CSC or whether they are a consequence of the disorder [7,78]. Therefore, one may wonder whether OSA and CSC patients are truly “physically stressed” because of their pathology or whether their high score on the DHS psychometric scale represents a self-oriented belief and is thus a mere consequence of the higher physical reactivity of these individuals [89].

## 5. Conclusions

The subtle dysfunctions of the HPA axis and the significant psychopathological and distress scores seen in both patients with OSA and CSC might constitute a common stress-related process linking these diseases. Future research should enlarge the population study by including women in order to evaluate if the pathophysiological mechanisms eventually linking stress with OSA and CSC might differ by sex.

However, the stressful condition induced by OSA itself may also be an independent risk factor for the development of OSA-induced CSCs. Furthermore, the mechanism by which stress hormone overactivity and allostatic overload could lead to the choroidal hyperpermeability seen in CSCs is still unclear [22,68,78].

Future research of our team will be focused on deepening the neuro-psycho-endocrinological aspects of OSA and CSC to evaluate the nature and significance of stress-related HPA axis derangements and to determine whether, in clinical practice, psychoeducation on effective coping strategies could help modify the course of the diseases and might be of value in the management of OSA and CSC patients.

## Figures and Tables

**Table 1:** Psychological profile in the study populations.

	Controls (*n* = 28)	OSA Patients (*n* = 14)	CSC Patients (*n* = 14)
HDS	4.0 ± 0.6 (2.2)	9.6 ± 0.4 (1.4) *	8.9 ± 1.6 (6.1) *
HAS	2.3 ± 0.5 (1.7)	5.4 ± 0.7 (2.8) *	2.3 ± 0.4 (1.5) #
DHS	73.9 ± 2.4 (9.0)	84.8 ± 3.2 (11.8) **	93.1 ± 4.0 (15.0) ***

Data are expressed as the mean ± SE (SD). List of abbreviations: HDS, Hamilton Depression Score; HAS, Hamilton Anxiety Score; DHS, Daily Hassles and Stress. Statistical Analysis: HDS: One-way ANOVA on Ranks; H = 27.435 with 2 degree of freedom (*p* < 0.001). Post-hoc test (Dunn’s method): * *p* < 0.05 vs. Controls. HAS: One-way ANOVA on Ranks; H = 18,577 with 2 degree of freedom (*p* < 0.001). Post-hoc test: * *p* < 0.05 vs. Controls; # *p* < 0.05 vs. OSA. DHS: One-way ANOVA: F_(2, 53)_ = 12,437 (*p* < 0.001). Post-hoc test (Holm–Sidak method): *** *p* < 0.001 (critical level 0.017); ** *p* = 0.008 (critical level 0.025).

**Table 2:** Salivary cortisol awake response (CAR) in the study populations.

Variable	Controls (*n* = 28)	OSA (*n* = 14)	CSC (*n* = 14)
Salivary Cortisol at awakening (ng/mL)	3.6 ± 0.2 (0.9)	4.0 ± 0.5 (1.9)	6.8 ± 0.4 (1.7) #
Salivary Cortisol at awakening + 30 min (ng/mL)	6.5 ± 0.4 (1.9) *	4.5 ± 0.2 (0.5) #	9.2 ± 0.6 (2.4) #, **

Data are shown as mean values ± SEM (SD). Statistical Analysis: Two Way repeated measures ANOVA: for salivary cortisol: factor GROUP F_(3, 111)_ = 22.475; *p* < 0.001; factor TIME F_(1, 111)_ = 77.507; *p* < 0.001; factor GROUP x TIME F_(3, 111)_ = 7.837; *p* = 0.001. Post hoc Bonferroni t-test for paired multiple comparisons: * *p* = 0.010, ** *p* < 0.001 respectively vs. awakening. Post-hoc Tukey’s test for all pairwise multiple comparison: # *p* < 0.05 vs. respective control time point.

**Table 3:** Salivary cortisol AUC_CAR_ and SLOPE_CAR_ in OSA and CSC study populations.

Variable	Controls (*n* = 28)	OSA Patients (*n* = 14)	CSC Patients (*n* = 14)
AUC_CAR_ (ng/mL/h)	5.1± 0.3 (1.1)	4.2± 0.2 (0.9)	7.5 ± 0.3 (1.9) * #
SLOPE_CAR_	6.0 ± 0.9 (3.2)	1.5 ± 0.9 (3.1) *	6.2 ± 1.3 (4.8) #

Data are expressed as the mean ± SE (SD). Statistical Analysis: AUC_CAR_: One-way ANOVA on Ranks; H = 28.005 with 2 degree of freedom (*p* < 0.001). Post-hoc test (Dunn’s method): * *p* < 0.05 vs. Controls; # *p* < 0.05 vs. OSA. SLOPE_CAR_: One-way ANOVA on Ranks; H = 10.543 with 2 degree of freedom (*p* < 0.005). Post-hoc test: (Dunn’s method): * *p* < 0.05 vs. Controls; # *p* < 0.05 vs. OSA.

## References

[1] Hiestand D.M., Britz P., Goldman M., Phillips B. (2006). Prevalence of symptoms and risk of sleep apnea in the US population: Results from the national sleep foundation sleep in America 2005 Poll. Chest.

[2] Jennum P., Riha R.L. (2009). Epidemiology of sleep apnoea/hypopnoea syndrome and sleep-disordered breathing. Eur. Respir. J..

[3] Young T., Peppard P.E., Gottlieb D.J. (2002). Epidemiology of obstructive sleep apnea: A population health perspective. Am. J. Respir. Crit. Care Med..

[4] Marin J.M., Carrizo S.J., Vicente E., Agusti A.G. (2005). Long-term cardiovascular outcomes in men with obstructive sleep apnoea-hypopnoea with or without treatment with continuous positive airway pressure: An observational study. Lancet.

[5] Grover D.P. (2010). Obstructive sleep apnea and ocular disorders. Curr. Opin. Ophthalmol..

[6] Glacet-Bernard A., Leroux L.J.G., Lasry S., Coscas G., Soubrane G., Souied E., Housset B. (2010). Obstructive sleep apnea among patients with retinal vein occlusion. Arch. Ophthalmol..

[7] Wu C.Y., Riangwiwat T., Rattanawong P., Nesmith B.L.W., Deobhakta A. (2018). Association of obstructive sleep apnea with central serous chorioretinopathy and choroidal thickness: A systematic review and meta-analysis. Retina.

[8] Maumenee A. (1965). Macular Diseases: Clinical Manifestations. Trans Am. Acad. Ophthalmol. Otolaryngol..

[9] Gass J.D. (1967). Pathogenesis of disciform detachment of the neuroepithelium: II. Idiopathic central serous choroidopathy. Am. J. Ophthalmol..

[10] Daruich A., Matet A., Dirani A., Bousquet E., Zhao M., Farman N., Jaisser F., Behar-Cohen F. (2015). Central Serous Chorioretinopathy: Recent Findings and New Physiopathology Hypothesis. Prog. Retin. Eye Res..

[11] Bernasconi P., Messmer E., Bernasconi A., Thölen A. (1998). Assessment of the sympatho-vagal interaction in central serous chorioretinopathy measured by power spectral analysis of heart rate variability. Graefes Arch. Clin. Exp. Ophthalmol..

[12] Kasasbeh E., Chi D.S., Krishnaswamy G. (2006). Inflammatory aspects of sleep apnea and their cardiovascular consequences (Review). South. Med. J..

[13] Michael J.C., Pack J., Pulido J., de Venecia G. (2003). Central serous chorioretinopathy associated with administration of sympathomimetic agents. Am. J. Ophthalmol..

[14] Somers V.K., Dyken M.E., Clary F.M.J. (1995). Sympathetic neural mechanisms in obstructive sleep apnea. J. Clin. Investig..

[15] Buckley T.M., Schatzberg A.F. (2005). On the interactions of the hypothalamic-pituitary-adrenal (HPA) axis and sleep: Normal HPA axis activity and circadian rhythm, exemplary sleep disorders. J. Clin. Endocrinol. Metab..

[16] Cortelli P., Lombardi C., Montagna P., Parati G. (2012). Baroreflex modulation during sleep and in obstructive sleep apnea syndrome. Auton. Neurosci..

[17] Späth-Schwalbe E., Gofferje M., Kern W., Born J., Fehm H.L. (1991). Sleep disruption alters nocturnal ACTH and cortisol secretory patterns. Biol. Psychiatry.

[18] Tomfohr L.M., Edwards K.M., Dimsdale J.E. (2012). Is obstructive sleep apnea associated with cortisol levels? A systematic review of the research evidence. Sleep Med. Rev..

[19] Henley D.E., Russell G.M., Douthwaite J.A., Wood S.A., Buchanan F., Gibson R., Woltersdorf W.W., Catterall J.R., Lightman S.L. (2009). Hypothalamic-pituitary-adrenal axis activation in obstructive sleep apnea: The effect of continuous positive airway pressure therapy. J. Clin. Endocrinol. Metab..

[20] Leveque T.Q., Yu L., Musch D.C., Chervin R.D., Zacks D.N. (2007). Central serous chorioretinopathy and risk for obstructive sleep apnea. Sleep Breath..

[21] Nicholson B.P., Atchison E., Idris A.A., Bakri S.J. (2018). Central serous chorioretinopathy and glucocorticoids: An update on evidence for association. Surv. Ophthalmol..

[22] Scarinci F., Ghiciuc C.M., Patacchioli F.R., Palmery M., Parravano M. (2019). Investigating the Hypothesis of Stress System Dysregulation as A Risk Factor for Central Serous Chorioretinopathy: A Literature Mini-Review. Curr. Eye Res..

[23] Schellevis R.L., van Dijk E.H.C., Breukink M.B., Altay L., Bakker B., Koeleman B.P.C., Kiemeney L.A., Swinkels D.W., Keunen J.E.E., Fauser S. (2018). Role of the Complement System in Chronic Central Serous Chorioretinopathy: A Genome-Wide Association Study. JAMA Ophthalmol..

[24] Imani M.M., Sadeghi M., Khazaie H., Emami M., Sadeghi Bahmani D., Brand S. (2020). Serum and Plasma Tumor Necrosis Factor Alpha Levels in Individuals with Obstructive Sleep Apnea Syndrome: A Meta-Analysis and Meta-Regression. Life.

[25] Kloos P., Laube I., Thoelen A. (2008). Obstructive sleep apnea in patients with central serous chorioretinopathy. Graefes Arch. Clin. Exp. Ophthalmol..

[26] Jain A.K., Kaines A., Schwartz S. (2010). Bilateral central serous chorioretinopathy resolving rapidly with treatment for obstructive sleep apnea. Graefes Arch. Clin. Exp. Ophthalmol..

[27] Huang Q.R., Qin Z., Zhang S., Chow C.M. (2008). Clinical patterns of obstructive sleep apnea and its comorbid conditions: A data mining approach. J. Clin. Sleep Med..

[28] Harris M., Glozier N., Ratnavadivel R., Grunstein R.R. (2009). Obstructive sleep apnea and depression. Sleep Med. Rev..

[29] Ohayon M. (2003). The effects of breathing-related sleep disorders on mood disturbances in the general population. J. Clin. Psychiatry.

[30] Sharafkhaneh A., Giray N., Richardson P., Young T., Hirshkowitz M. (2005). Association of psychiatric disorders and sleep apnea in a large cohort. Sleep.

[31] Vandeputte M., Weerd A. (2003). Sleep disorders and depressive feelings: A global survey with the Beck depression scale. Sleep Med..

[32] Hayashida K., Inoue Y., Chiba S., Yagi T., Urashima M., Honda Y., Itoh H. (2007). Factors influencing subjective sleepiness in patients with obstructive sleep apnea syndrome. Psychiatry Clin. Neurosci..

[33] Pierobon A., Giardini A., Fanfulla F., Callegari S., Majani G. (2008). A multidimensional assessment of obese patients with obstructive sleep apnoea syndrome (OSAS): A study of psychological, neuropsychological and clinical relationships in a disabling multifaceted disease. Sleep Med..

[34] Siguan C.S., Aguilar R.N. (2014). Psychological profile of patients with central serous retinopathy. Philipp. J. Ophthalmol..

[35] Piskunowicz M., Jaracz M., Lesiewska H., Malukiewicz G., Brozek-Pestka M., Borkowska A. (2014). Temperament profile in patients with central serous chorioretinopathy: A case-control study. Eur. J. Ophthalmol..

[36] Bazzazi N., Ahmadpanah M., Akbarzadeh S., Seif Rabiei M.A., Holsboer-Trachsler E., Brand S. (2015). In patients suffering from idiopathic central serous chorioretinopathy, anxiety scores are higher than in healthy controls, but do not vary according to sex or repeated central serous chorioretinopathy. Neuropsychiatr. Dis. Treat..

[37] Spahn C., Wiek J., Burger T., Hansen L. (2003). Psychosomatic aspects in patients with central serous chorioretinopathy. Br. J. Ophthalmol..

[38] Conrad R., Bodeewes I., Schilling G., F Geiser F., Imbierowicz K., Liedtke R. (2000). Central serous chorioretinopathy and psychological stress. Ophthalmologe.

[39] Conrad R., Geiser F., Kleiman A., Zur B., Karpawitz-Godt A. (2014). Temperament and character personality profile and illness-related stress in central serous chorioretinopathy. Sci. World J..

[40] Mansour A.M., Hamam R. (2017). Operating room central serous chorioretinopathy. SAGE Open Med. Case. Rep..

[41] Mansuetta C.C., Mason J.O., Swanner J., Feist R.M., White M.F., Thomley M.L., McGwin G., Emond T.L. (2004). An association between central serous chorioretinopathy and gastroesophageal reflux disease. Am. J. Ophthalmol..

[42] Hamilton M. (1960). A rating scale for depression. J. Neurol. Neurosurg. Psychiatry..

[43] Hamilton M. (1959). The assessment of anxiety states by rating. Br. J. Med. Psychol..

[44] Kohn P.M., Macdonald J.E. (1992). The Survey of Recent Life Experiences: A decontaminated Hassles scale for adults. J. Behav. Med..

[45] Rosner B. (2011). Fundamentals of Biostatistics.

[46] Frankenfield D.C., Rowe W.A., Cooney R.N., Smith J.S., Becker D. (2001). Limits of Body Mass Index to Detect Obesity and Predict Body Composition. Nutrition.

[47] ESC /ESH Hypertension Guidelines Committee (2007). Guidelines for the management of arterial hypertension: The Task Force for the Management of Arterial Hypertension of the European Society of Hypertension (ESH) and of the European Society of Cardiology (ESC). Eur. Heart. J..

[48] Tittl M.K., Spaide R.F., Wong D., Pilotto E., Yannuzzi L.A., Fisher Y.L., Freund B., Guyer D.R., Slakter J.S., Sorenson J.A. (1999). Systemic findings associated with central serous chorioretinopathy. Am. J. Ophthalmol..

[49] Spaide R.F., Campeas L., Haas A., Yannuzzi L.A., Fisher Y.L., Guyer D.R., Slakter J.S., Sorenson J.A., Orlock D.A. (1996). Central serous chorioretinopathy in younger and older adults. Ophthalmology.

[50] Spaide R.F., Hall L., Haas A., Campeas L., Yannuzzi L.A., Fisher Y.L., Guyer D.R., Slakter J.S., Sorenson J.A., Orlock D.A. (1996). Indocyanine green video angiography of older patients with central serous chorioretinopathy. Retina.

[51] Regillo C.D., Benson W.E., Maguire J.I., Annesley W.H. (1994). Indocyanine green angiography and occult choroidal neovascularization. Ophthalmology.

[52] Hackney A.C., Viru A. (2008). Research methodology: Endocrinology measurements in exercise science and sports medicine. J. Athl. Train..

[53] Simeoni S., Biselli R., D’Amelio R., Rocca B., Lattanzio S., Mucci L., Davì G., Patacchioli F.R. (2011). Stress-induced salivary cortisol secretion during hypobaric hypoxia challenge and in vivo urinary thromboxane production in healthy male subjects. Stress.

[54] Pruessner J.C., Wolf O.T., Hellhammer D.H., Buske-Kirschbaum A., von Auer K., Jobst S., Kaspers F., Kirschbaum C. (1997). Free cortisol levels after awakening: A reliable biological marker for the assessment of adrenocortical activity. Life Sci..

[55] Fekedulegn D.B., Andrew M.E., Burchfiel C.M., Violanti J.M., Hartley T.A., Charles L.E., Miller D.B. (2007). Area under the curve and other summary indicators of repeated waking cortisol measurements. Psychosom. Med..

[56] Winer B.J. (1970). Statistical Principles in Experimental Design.

[57] Clow A., Thorn L., Evans P., Hucklebridge F. (2004). The awaking cortisol response: Methodological issue and significant. Stress.

[58] Bhattacharyya M.R., Molloy G.J., Steptoe A. (2008). Depression is associated with flatter cortisol rhythms in patients with coronary artery disease. J. Psychosom. Res..

[59] Vreeburg S.A., Hartman C.A., Hoogendijk W.J., van Dyck R., Zitman F.G., Ormel J., Penninx B.W. (2010). Parental history of depression or anxiety and the cortisol awakening response. Br. J. Psychiatry.

[60] Delle Chiaie R., Trabucchi G., Girardi N., Marini I., Vergnani L., Caredda M., Zerella M.P., Minichino A., Patacchioli F.R., Simeoni S. (2013). Group psychoeducation normalizes cortisol awakening response in stabilized bipolar patients under pharmacological maintenance treatment. Psychother. Psychosom..

[61] Wessa M., Rohleder N., Kirschbaum C., Flor H. (2006). Altered cortisol awakening response in posttraumatic stress disorder. Psychoneuroendocrinology.

[62] Sjörs A., Ljung T., Jonsdottir I.H. (2012). Long-term follow-up of cortisol awakening response in patients treated for stress-related exhaustion. BMJ.

[63] Nater U.M., Maloney E., Boneva R.S., Gurbaxani B.M., Lin J.M., Jones J.F., Reeves W.C., Heim C. (2008). Attenuated morning salivary cortisol concentrations in a population-based study of persons with chronic fatigue syndrome and well controls. J. Clin. Endocrinol. Metab..

[64] Bauer A.M., Quas J.A., Boyce W.T. (2002). Associations between physiological reactivity and children’s behavior: Advantages of a multisystem approach. J. Dev. Behav. Pediatrics.

[65] Chrousos G.P. (2009). Stress and disorders of the stress system. Nat. Rev. Endocrinol..

[66] Cozma S., Ghiciuc C.M., Damian L., Pasquali V., Saponaro A., Lupusoru E.C., Patacchioli F.R., Dima-Cozma L.C. (2018). Distinct activation of the sympathetic adreno-medullar system and hypothalamus pituitary adrenal axis following the caloric vestibular test in healthy subjects. PLoS ONE.

[67] Stetler C., Miller G.E. (2011). Depression and hypothalamic-pituitary-adrenal activation: A quantitative summary of four decades of research. Psychosom. Med..

[68] McEwen B.S. (2003). Mood disorders and allostatic load. Biol. Psychiatry.

[69] Vigil J.M., Geary D.C., Granger D.A., Flinn M.V. (2010). Sex differences in salivary cortisol, alpha-amylase, and psychological functioning following hurricane katrina. Child Dev..

[70] Ali N., Pruessner J.C. (2012). The salivary alpha amylase over cortisol ratio as a marker to assess dysregulations of the stress systems. Physiol. Behav..

[71] Raff H., Ettema S.L., Eastwood D.C., Woodson B.T. (2011). Salivary cortisol in obstructive sleep apnea: The effect of CPAP. Endocrine.

[72] Carneiro G., Togeiro S.M., Hayashi L.F., Ribeiro-Filho F.F., Ribeiro A.B., Tufik S., Zanella M.T. (2008). Effect of continuous positive airway pressure therapy on hypothalamic-pituitary-adrenal axis and 24h blood pressure in men with obstructive sleep apnea syndrome. Am. J. Physiol. Endocrinol. Metab..

[73] Dadoun F., Darmon P., Achard V., Boullu C., Philip-Joet F., Alessi M.C., Rey M., Grino M., Dutour A. (2007). Effect of sleep apnea syndrome on the circadian profile of cortisol in obese men. Am. J. Physiol. Endocrinol. Metab..

[74] Vgontzas S., Pejovic E., Zoumakis H.M., Lin E.O., Bixler M., Basta J., Fang A., Sarrigiannidis A., Chrousos G.P. (2007). Daytime napping after a night of sleep loss decreases sleepiness, improves performance, and causes beneficial changes in cortisol and interleukin-6 secretion. Am. J. Physiol. Endocrinol. Metab..

[75] Wirtz P.H., von Kanel R., Emini L., Ruedisueli K., Groessbauer S., Maercker A., Ehlert U. (2007). Evidence for altered hypothalamus-pituitary-adrenal axis functioning in systemic hypertension: Blunted cortisol response to awakening and lower negative feedback sensitivity. Psychoneuroendocrinology.

[76] Rosmond R., Dallman M.F., Björntorp P. (1998). Stress-related cortisol secretion in men: Relationships with abdominal obesity and endocrine, metabolic and hemodynamic abnormalities. J. Clin. Endocrinol. Metab..

[77] Whitworth J.A., Brown M.A., Kelly J.J., Williamson P.M. (1995). Mechanisms of cortisol-induced hypertension in humans. Steroids.

[78] Kumar M., van Dijk E.H.C., Raman R., Mehta P., Boon C.J.F., Goud A., Bharani S., Chhablani J. (2020). Stress and Vision-Related Quality of Life in Acute and Chronic Central Serous Chorioretinopathy. BMC Ophthalmol..

[79] Chida Y., Steptoe A. (2009). Cortisol awakening response and psychosocial factors: A systematic review and meta-analysis. Biol. Psychol..

[80] Kaplan R. (1992). Obstructive sleep apnoea and depression: Diagnostic implications. Aust. N. Z. J. Psychiatry.

[81] Millman R.P., Fogel B.S., McNamara M.E., Carlisle C.C. (1989). Depression as a manifestation of obstructive sleep apnea: Reversal with nasal continuous positive airway pressure. J. Clin. Psychiatry.

[82] Mosko S.S., Zetin M., Glen S., Garber D., DeAntonio M., Sassin J., McAnich J., Warren S. (1989). Self-reported depressive symptomatology, mood ratings, and treatment outcome in sleep disorders patients. J. Clin. Psychol..

[83] Reynolds C.F., Kupfer D.J., McEachran A.B., Taska L.S., Sewitch D., Coble P.A. (1984). Depressive psychopathology in male sleep apneics. J. Clin. Psychiatry.

[84] Peppard P.E., Szklo-Coxe M., Hla K.M., Young T. (2006). Longitudinal association of sleep-related breathing disorder and depression. Arch. Intern. Med..

[85] Chen Y.-H., Keller J.K., Kang J.-H., Hsieh H.-J., Lin H.-C. (2013). Obstructive sleep apnea and the subsequent risk of depressive disorder: A population-based follow-up study. J. Clin. Sleep Med. JCSM.

[86] Nanthakumar S., Bucks R.S., Skinner T.C., Starkstein S., Hillman D., James A., Hunter M. (2017). Assessment of the Depression, Anxiety, and Stress Scale (DASS-21) in untreated obstructive sleep apnea (OSA). Psychol. Assess..

[87] Caccavale A., Romanazzi F., Imparato M., Negri A., Morano A., Ferentini F. (2011). Central serous chorioretinopathy: A pathogenetic model. Clin. Ophthalmol..

[88] Hellhammer J., Fries E., Schweisthal O.W., Schlotz W., Stone A.A., Hagemann D. (2007). Several daily measurements are necessary to reliably assess the cortisol rise after awakening: State and trait components. Psychoneuroendocrinology.

[89] Imani M.M., Sadeghi M., Khazaie H., Emami M., Sadeghi Bahmani D., Brand S. (2020). Evaluation of serum and plasma interleukin-6 levels in obstructive sleep apnea syndrome (OSAS): A meta-analysis and meta-regression. Front. Immunol..

